# A Structural Systems Biology Approach to High-Risk CG23 Klebsiella pneumoniae

**DOI:** 10.1128/mra.01013-22

**Published:** 2023-01-25

**Authors:** Nicole L. Inniss, Travis J. Kochan, George Minasov, Zdzislaw Wawrzak, Changsoo Chang, Kemin Tan, Ludmilla Shuvalova, Olga Kiryukhina, Sergii Pshenychnyi, Ruiying Wu, Ivegeniia Dubrovska, Gyorgy Babnigg, Michael Endres, Wayne F. Anderson, Alan R. Hauser, Andrzej Joachimiak, Karla J. F. Satchell

**Affiliations:** a Department of Microbiology-Immunology, Feinberg School of Medicine, Northwestern University, Chicago, Illinois, USA; b Center for Structural Biology of Infectious Diseases, Feinberg School of Medicine, Northwestern University, Chicago, Illinois, USA; c Northwestern Synchrotron Research Center, Life Sciences Collaborative Access Team (LS-CAT), Northwestern University, Argonne, Illinois, USA; d Structural Biology Center, X-ray Science Division, Argonne National Laboratory, Lemont, Illinois, USA; e Recombinant Protein Production Core, Northwestern University, Evanston, Illinois, USA; f Department of Biochemistry and Molecular Genetics, Feinberg School of Medicine, Northwestern University, Chicago, Illinois, USA; g Division of Infectious Diseases, Department of Medicine, Feinberg School of Medicine, Northwestern University, Chicago, Illinois, USA; Indiana University, Bloomington

## Abstract

Klebsiella pneumoniae is a leading cause of antibiotic-resistant-associated deaths in the world. Here, we report the deposition of 14 structures of enzymes from both the core and accessory genomes of sequence type 23 (ST23) K1 hypervirulent K. pneumoniae.

## ANNOUNCEMENT

Klebsiella pneumoniae is a common commensal of the gastrointestinal tract but can cause opportunistic and severe infections like pneumonia, urinary tract infections, and bacteremia (1). With the pervasiveness of multidrug-resistant classical strains of K. pneumoniae (cKp) and the ongoing emergence of hypervirulent K. pneumoniae (hvKp), such as sequence type 23 (ST23) strains, the infectious disease community anticipates an increase in the convergence of hypervirulence and carbapenem resistance, which will yield K. pneumoniae strains that are highly virulent and exceptionally difficult to treat (2, 3). *K. pneumoniae* is now one of the top six leading causes of antibiotic-resistance associated deaths (4). As K. pneumoniae is rapidly evolving to evade conventional antibiotic treatment, it is vital to develop novel therapeutics to improve the outcomes of patients infected with these strains. The Center for Structural Biology of Infectious Diseases (CSBID) established a structure determination pipeline for proteins that are associated with high-risk ST23 *Κ. pneumoniae* isolates (5). These proteins represent targets that are potentially amenable to structure-based drug design geared toward treating K. pneumoniae infections. Targets were selected using a shotgun approach aimed at uncharacterized metabolic proteins from ST23 K1 hypervirulent K. pneumoniae. A total of 196 proteins were determined to be amenable to protein crystallization and were admitted into the pipeline. To categorize the selected genes, all available K. pneumoniae assemblies were downloaded from the National Center for Biotechnology Information in July 2020 (*n* = 8,182). K. pneumoniae assemblies were searched for gene targets using BLAST (v2.9.0+) and considered present if the target had a minimum sequence identity of 85% and minimum total alignment of 90% using a custom Python script (Python 3.7.4). *In silico* multilocus sequence typing of all K. pneumoniae isolates was completed using *Kleborate* (6). This analysis showed that 64 of the selected proteins are part of the K. pneumoniae core genome, defined as genes present in at least 95% of all strains; the other 132 proteins are part of the K. pneumoniae accessory genome and are overrepresented in the highly virulent hvKp clonal group CG23, which contains ST23 (7).

The genes encoding the selected 196 uncharacterized proteins from the K. pneumoniae core and accessory genomes were amplified by PCR using genomic DNA as a template and primers. Based on homology to previously solved structures and predicted crystallizability (8), full genes and/or small truncations for the targets were amplified. The PCR products were cloned into plasmids pMCSG53, pMCSG68, and pMCSG73 (Protein Structure Initiative [PSI]:Biology-Materials Repository, http://psimr.asu.edu) according to the ligation-independent cloning procedure (9, 10). The various vectors introduce protease-cleavable solubility tags in addition to a six-histidine (His_6_) purification tag at the N or C terminus of the expressed proteins. The resulting clones were transformed into T7-polymerase-expressing Escherichia coli strains and were tested in small scale for expression, solubility, and purification efficiency. Plasmids that expressed soluble proteins were sequenced at the University of Chicago Cancer Research DNA Sequencing Facility.

All targets that were expressed successfully in small scale were purified at large scale according to previously published protocols (11, 12). Briefly, transformed E. coli bacteria cultured in either Terrific broth or selenomethione containing minimal medium with antibiotics were grown to an optical density at 600 nm (OD_600_) of 0.5 to 1.0 and induced with 1 mM isopropyl-β-d-thio-galactopyranoside at 18°C or 25°C overnight. Cells were harvested by centrifugation, resuspended in lysis buffer, and lysed by the freeze-thaw method followed by sonication. Lysates were clarified by centrifugation at 30,000 × *g*, filtered at 0.45 or 0.22 μM, and loaded onto 5-mL HiTrap chelating high-performance (HP) or HiTrap fast flow (FF) columns (GE Healthcare Life Sciences/Cytiva) using ÄKTA fast-proteins liquid chromatography systems (GE Healthcare Life Sciences/Cytiva). Eluted proteins from targets yielding structures with Research Collaboratory for Structural Bioinformatics (RCSB) Protein Data Bank (PDB) codes 6X1L and 7TL5 were applied immediately to a HiPrep 26/10 desalting column (GE Healthcare Life Sciences/Cytiva) for buffer exchange. Eluted proteins from all other targets that yielded structures were immediately applied to a Superdex 100 26/600 column (GE Healthcare Life Sciences/Cytiva). All purified proteins were incubated with tobacco etch virus (TEV) protease that was purified with a His_6_ tag at a 1:20 or 1:30 TEV:protein ratio during overnight dialysis at room temperature, and the cleaved proteins were collected in the flow through following passage over an Ni-nitrilotriacetic acid (NTA) affinity column. Samples were analyzed for purity using SDS-PAGE, concentrated, and set up in crystallization trials.

Concentrated proteins were set up as 2-μL crystallization drops in 96-well plates using commercially available and in-house crystallization screens. Crystals deemed suitable for screening were cryoprotected, frozen, and then screened for data collection at the Structural Biology Center and the Life Sciences-Collaborative Access Team at the Advanced Photon Source (APS), Argonne National Laboratory. Structures of proteins grown in selenomethionine medium were solved by the single-wavelength anomalous diffraction method (SAD), using the automatic structure solution from HKL-3000 (13) and autobuild package from PHENIX (14). Structures of native proteins were solved by molecular replacement (MR) using PHASER, MORDA, and MRBUMP from the CCP4 suite (15). Structures were refined using REFMAC5 (16) or PHENIX and visually corrected in Coot (17). Water molecules were generated using ARP/wARP (18), ligands were fit into electron density maps in Coot. Translation-libration-screw (TLS) groups were generated by the TLS motion determination (TLSMD) server (19), and corrections were applied at the final steps of refinement. Models were validated using MolProbity (20), and coordinates of the final models and experimental data were deposited to the PDB.

**FIG 1:**
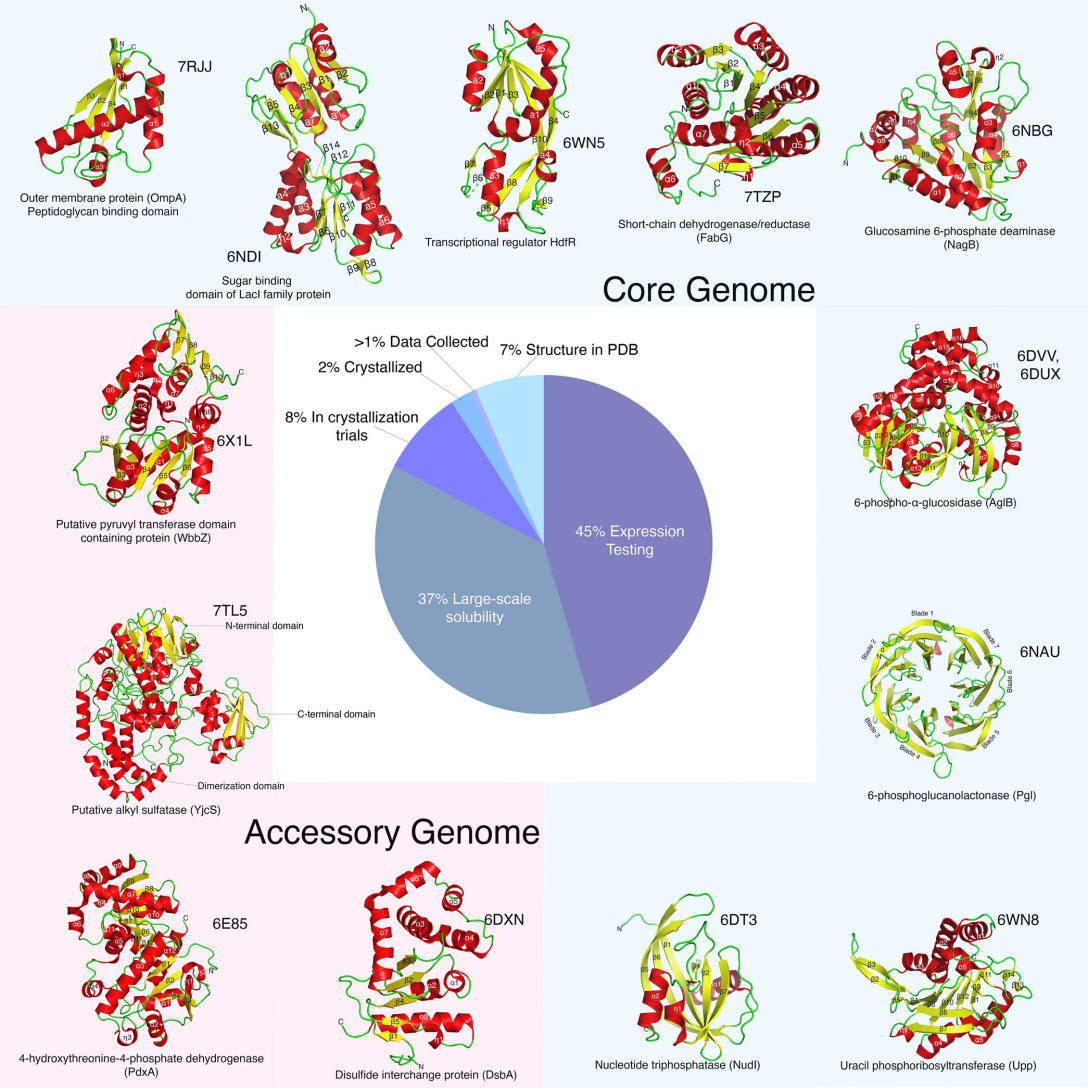
Status of Klebsiella pneumoniae pipeline targets. Structures of proteins deposited with the indicated PDB codes for 13 targets from the K. pneumoniae structure determination pipeline are depicted as cartoons with α-helices colored red, β-strands colored yellow, and loops colored lime green. Proteins found in the core genome are framed in light blue and those derived from the accessory genome are in pink. The pie chart details the percentage of targets currently at each stage in the pipeline.

In total, 73 of the 196 targets were determined soluble after cloning and small-scale expression and solubility testing. Of these targets, 34 were successfully purified from large-scale preparations and entered crystallization trials. Fourteen structures were determined for 13 different targets as detailed in Fig. 1. Overall, the success rate from target selection through structure determination was just below 7.0%. The crystal structures resulting from this K. pneumoniae structural genomics pipeline have been deposited in the PDB. Refinement statistics for each deposit are listed in Table 1, and all details on data quality are available on the PDB. As standard practice, targets for which structures were not solved and were not deemed as “high priority” to the requester did not undergo further optimization for expression, purification, or crystallization. These “stalled” targets are archived until a new request for structure determination is proposed.

**TABLE 1:** Data quality and refinement statistics

Parameter	Data by PDB accession code^*a*^													
	6nbg	6nau	6dt3	6wn8	6dvv	6dux	6ndi	6wn5	6dxn	6e85	7rjj	7tzp	7tl5	6x1l
Data collection														
Space group	*C222_1_*	*P6_2_*	*P1*	*P3_1_21*	*P3_2_21*	*P1*	*C2*	*P4_3_2_1_2*	*P1*	*C2*	*P6_1_*	*F222*	P2_1_2_1_2_1_	H32
Unit cell parameters														
Edges (Å)														
*a*	75.48	184.75	33.14	203.35	86.03	85.23	180.74	62.39	42.4	97.36	41.89	161.39	76.45	104.2
*b*	123.81	184.75	39.35	203.35	86.03	85.23	110.48	62.39	42.39	76.22	41.89	256.02	80.16	104.2
*c*	299.66	48.76	56.29	157.78	230.78	228.86	37.78	103.13	103.95	92.11	267.93	260.64	243.83	194.5
Angles (°)														
*α*	90.00	90.00	77.07	90.00	90.00	77.07	90.00	90.00	90.27	90.00	90.00	90.00	90.00	90.00
*β*	90.00	90.00	80.74	90.00	90.00	80.74	97.94	90.00	89.78	106.78	90.00	90.00	90.00	106.78
*γ*	90.00	120.00	86.20	120.00	120.00	86.20	90.00	90.00	96.00	90.00	120.00	90.00	90.00	120.00
Resolution range (Å)	30.00–2.05 (2.09–2.05)	30.00–1.55 (1.58–1.55)	30.00–1.20 (1.22–1.20)	30.00–2.70 (2.75–2.70)	30.00–2.25 (2.29–2.25)	30.00–2.25 (2.29–2.25)	30.00–2.60 (2.64–2.60)	30.00–1.52 (1.55–1.52)	30.00–1.95 (1.98–1.95)	30.00–1.25 (1.27–1.25)	30.00–1.88 (1.91–1.88)	30.00–2.60 (2.64–2.60)	50–2.70 (2.75–2.7)	41.00–2.00 (2.03–2.00)
No. of reflections	87,989 (4,325)	138,516 (6,923)	78,810 (3,881)	97,876 (5,133)	47,795 (2,327)	46,596 (2,254)	22,623 (1,122)	32,133 (1,569)	51,946 (2,517)	177,342 (8,637)	21,507 (1,087)	82,427 (4,121)	38,419 (1,662)	27,259 (1,163)
*R*_merge_ (%)	9.5 (83.2)	10.0 (71.1)	7.0 (79.0)	9.0 (79.0)	9.2 (83.3)	10.2 (78.2)	11.4 (80.4)	5.7 (78.3)	13.5 (78.6)	5.3 (54.9)	7.3 (135.8)	15.7 (122.9)	6.4 (100.8)	7.4 (68.5)
Completeness (%)	99.8 (100.0)	100.0 (100.0)	92.3 (91.1)	100.0 (100.0)	99.7 (99.5)	100.0 (100.0)	99.9 (100.0)	100.0 (100.0)	97.8 (96.3)	99.8 (97.7)	99.9 (100.0)	100.0 (100.0)	90.4 (79.4)	98.6 (85.6)
〈*I*/*σ*(*I*)〉	20.5 (2.0)	20.5 (3.2)	13.7 (2.1)	20.3 (2.4)	25.2 (2.5)	22.9 (3.0)	19.3 (2.3)	44.4 (3.4)	16.8 (2.7)	24.6 (2.3)	31.5 (1.8)	13.8 (2.0)	20.1 (1.4)	34.4 (1.3)
Multiplicity	5.5 (5.5)	9.8 (8.5)	2.8 (2.9)	6.6 (6.7)	7.4 (7.5)	8.5 (8.6)	6.3 (6.4)	9.5 (9.5)	4.4 (4.3)	4.3 (3.3)	11.4 (10.5)	7.6 (7.7)	5.1 (4.9)	5.2 (4.5)
Wilson *B* factor	31.6	12.8	12.3	55.2	46.1	40.2	55.2	22.2	18.5	12.3	17.3	43	39.4	52.8
Refinement														
Resolution range (Å)	29.62–2.05 (2.10–2.05)	29.33–1.55 (1.59–1.55)	27.14–1.20 (1.23–1.20)	29.71–2.70 (2.77–2.70)	28.99–2.25 (2.31–2.25)	29.89–2.22 (2.31–2.25)	29.83–2.60 (2.67–2.60)	29.86–1.52 (1.56–1.52)	27.01–1.95 (1.99–1.95)	29.67–1.25 (1.28–1.25)	28.16–1.88 (1.93–1.88)	29.99–2.60 (2.67–2.60)	41.66–2.70 (2.73–2.70)	41.00–2.00 (2.07–2.00)
Completeness (%)	99.5 (96.3)	100.0 (100.0)	92.2 (89.7)	99.9 (99.6)	99.7 (99.5)	99.8 (99.1)	99.7 (98.4)	99.9 (99.9)	96.4 (78.2)	99.8 (97.7)	99.9 (99.9)	99.9 (99.8)	82.2 (36.0)	98.2 (89.0)
No. of reflections	83,398 (6,211)	131,376 (10,167)	74,887 (5,692)	83,398 (7,489)	45,255 (3,451)	44,168 (3,363)	21,591 (1,645)	30,411 (2,314)	48,932 (3,011)	168,266 (12,805)	19,057 (588)	78,066 (5,996)	35,056 (1,217)	27,157 (2,430)
*R*_work_/*R*_free_ (%)	20.1/23.1 (29.7/32.1)	12.6/15.6 (18.9/21.5)	15.3/18.7 (23.7/25.0)	18.9/21.9 (29.1/30.8)	16.4/21.9 (23.4/27.1)	15.4/19.1 (21.4/26.6)	18.2/23.5 (31.3/32.9)	18.2/20.5 (24.1/27.4)	18.0/23.0 (17.7/23.9)	11.4/13.9 (22.5/24.0)	20.1/22.8 (24.0/27.4)	18.9/22.7 (28.5/31.8)	20.9 (24.8)	19.1/22.1 (33.4/34.7)
Protein chains/atoms	6/11,006	3/7,698	3/2,312	10/15,854	2/6,816	2/6,807	2/4,216	1/1,399	4/6,032	2/4,923	2/1,848	8/14,442	2/9,851	1/2,080
Ligand/solvent atoms	41/763	83/1,779	15/343	561/473	118/370	122/359	0/157	3/193	20/365	38/954	14/178	344/700	4/75	1/32
Mean temp factor (Å^2^)	41.5	12.5	16.8	55.6	59.3	49	58.8	26.8	22.9	16.9	22.8	49.1	46	65.5
Coordinate deviations														
RMSD bonds (Å)	0.006	0.008	0.008	0.002	0.008	0.007	0.005	0.008	0.006	0.006	0.006	0.003	0.002	0.003
RMSD angles (°)	1.469	1.376	1.4	1.167	1.392	1.365	1.395	1.499	1.133	1.283	1.442	1.175	0.511	0.521
Ramachandran plot														
Favored (%)	96	97	98	95	96	98	94	98	94	98	99	95	94.4	94.5
Allowed (%)	4	2	2	5	4	2	5	2	6	2	1	5	5.2	4.6
Outside allowed (%)	0	1	0	0	0	0	1	0	0	0	0	0	0.4	0.9

a Values in parentheses are for the outer shell.

In all, structures represent both metabolic proteins and possible transcriptional regulators. Nine structures are of core genome proteins, of which one was previously annotated as a “hypothetical protein” but has structural similarity to glucosamine 6-phosphate deaminase (PDB code 6NBG). Four structures are of accessory genome proteins, of which one was also annotated as a hypothetical protein but has structural similarity to pyruvyl transferase (PDB code 6X1L). Lastly, only one structure (PDB code 7TZP) is of a protein with published functional data (21). Thus, the x-ray crystal structures presented here provide the basis for functional analysis of 12 uncharacterized proteins associated with high-risk K. pneumoniae infection.

### Data availability.

The details of primers used for full-length and/or truncated expression constructs and the number of expression constructs generated for each target are available online at https://csgid.org/targets/index listed as batches “set388,” “set389,” and “set468.” The status of each target in the pipeline is also provided. Details of minor protocol modifications for expression and purification of each independent target are available online at http://csgid.org and/or by request sent to the Center for Structural Biology of Infectious Diseases. All coordinates for all final models and experimental data have been deposited to the Protein Data Bank (https://www.rcsb.org/), and can be found using PDB codes 6nbg, 6nau, 6dt3, 6wn8, 6dvv, 6dux, 6ndi, 6wn5, 6dxn, 6e85, 7rjj, 7tzp, 7tl5, and 6x1l.
